# Current concepts: tissue engineering and regenerative medicine applications in the ankle joint

**DOI:** 10.1098/rsif.2013.0784

**Published:** 2014-03-06

**Authors:** S. I. Correia, H. Pereira, J. Silva-Correia, C. N. Van Dijk, J. Espregueira-Mendes, J. M. Oliveira, R. L. Reis

**Affiliations:** 1 3B's Research Group—Biomaterials, Biodegradables and Biomimetics, University of Minho, Headquarters of the European Institute of Excellence on Tissue Engineering and Regenerative Medicine, AvePark, S. Cláudio de Barco, Taipas, Guimarães 4806-909, Portugal; 2 ICVS/3B's—PT Government Associate Laboratory, Braga/Guimarães, Portugal; 3 Saúde Atlântica Sports Center—F.C. Porto Stadium, Minho University and Porto University Research Center, Porto, Portugal; 4 Orthopedic Department, Centro Hospitalar Póvoa de Varzim, Vila do Conde, Portugal; 5 Orthopedic Department, Amsterdam Medical Centre, Amsterdam, The Netherlands

**Keywords:** ankle, biomaterials, osteochondral lesions, regenerative medicine, scaffold, tissue engineering

## Abstract

Tissue engineering and regenerative medicine (TERM) has caused a revolution in present and future trends of medicine and surgery. In different tissues, advanced TERM approaches bring new therapeutic possibilities in general population as well as in young patients and high-level athletes, improving restoration of biological functions and rehabilitation. The mainstream components required to obtain a functional regeneration of tissues may include biodegradable scaffolds, drugs or growth factors and different cell types (either autologous or heterologous) that can be cultured in bioreactor systems (*in vitro*) prior to implantation into the patient. Particularly in the ankle, which is subject to many different injuries (e.g. acute, chronic, traumatic and degenerative), there is still no definitive and feasible answer to ‘conventional’ methods. This review aims to provide current concepts of TERM applications to ankle injuries under preclinical and/or clinical research applied to skin, tendon, bone and cartilage problems. A particular attention has been given to biomaterial design and scaffold processing with potential use in osteochondral ankle lesions.

## 1. Introduction: fundamentals of tissue engineering and regenerative medicine

### 1.1. Tissue engineering and regenerative medicine surgical application potential in several ankle tissues

In the anatomical ankle region, several tissues develop injuries/pathologies with new emerging therapeutic possibilities arising from tissue engineering and regenerative medicine (TERM) strategies.

Tissue engineering (TE) and related therapeutic strategies, which mimic the mechanisms of tissue normal repair and regeneration, have been regarded as a revolution in medical sciences [1]. As stated by Langer & Vacanti [1], TE is the research field which combines the principles of engineering, and life and health sciences with the development of biological functional substitutes. The aim is to restore, defend (avoid disease progression) or improve the function of the damaged tissue and/or organ.

**Figure 1.**
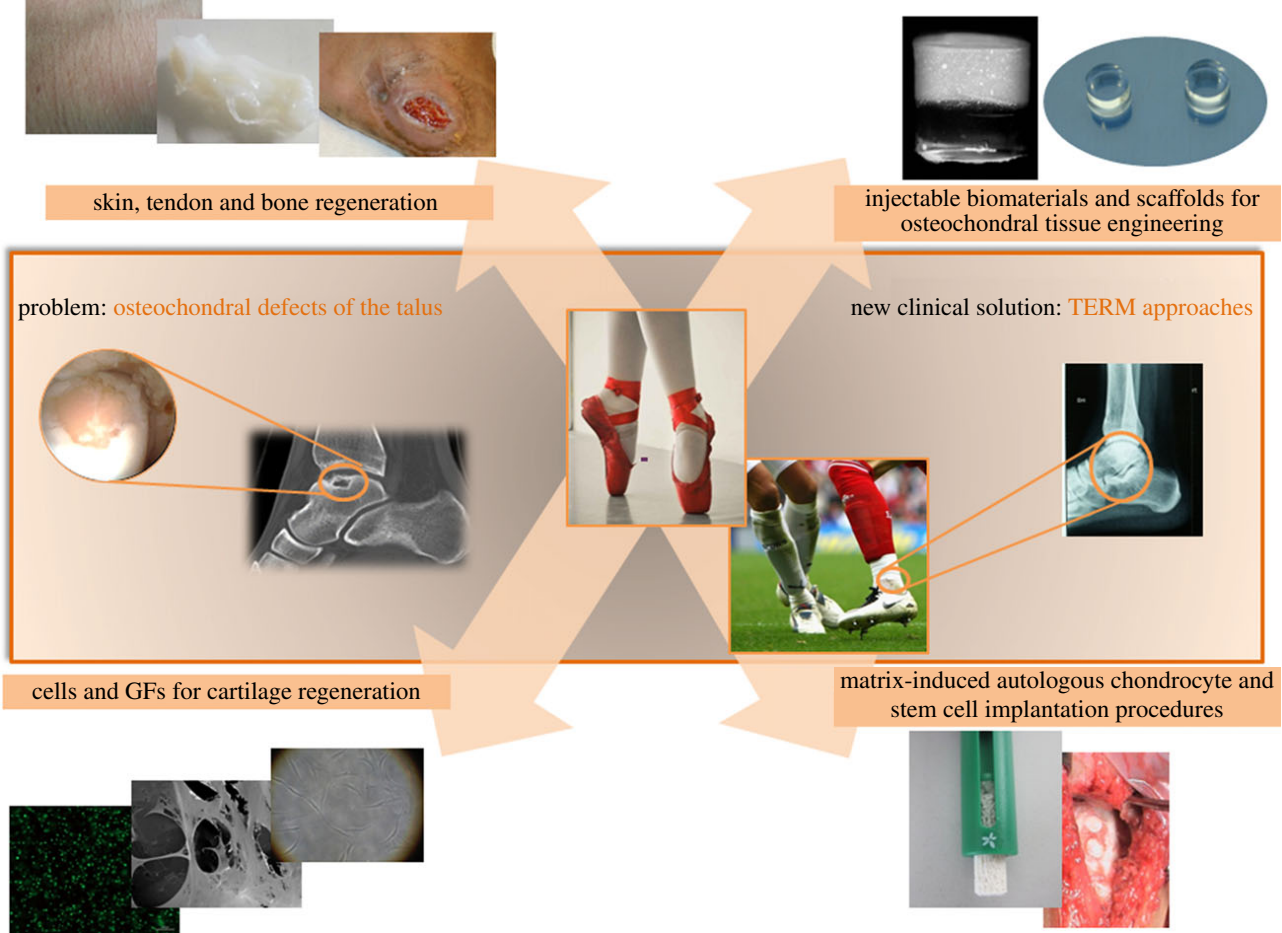
TERM applications on the ankle joint.

Application of ankle TE strategies [2,3] can consider, by definition, three main variables (figure 1): (i) tridimensional porous supports or scaffolds [4,5], (ii) cells (differentiated or undifferentiated), and (iii) bioactive agents, i.e. physical stimulus [6], and/or growth factors (GFs) [7,8]. Cells can be seeded and cultured onto a structure or scaffold capable of supporting three-dimensional tissue formation [9]. GFs can be used in the isolated form in injured tissue/organ, as a ‘pool’ of GFs or in association with scaffolds and/or cells [10,11]. The use of bioreactors (dynamic systems) as a way to improve the *in vitro* biological and mechanical properties of the TE constructs (cell-laden scaffolds) is also advantageous, as it can allow one to overcome the limitation of nutrients/metabolites diffusion observed in static cultures [12]. On the other hand, regenerative medicine (RM) is a broader concept which, besides that previously discussed for TE, also considers the use of bioactive soluble molecules [13,14], stem cell technologies [15,16], prolotherapy (i.e. injectable regenerative techniques) [17], genetic therapeutic strategies [18], nanotechnologies and several medical devices.

The terms TE and RM can be used interchangeably, but both fields have been globally referred to in association as TERM [9,19].

In this review, an overview is given of the present applications in treatment of skin, tendon, bone and osteochondral lesions in the ankle joint.

#### 1.1.1. Applications of tissue engineering and regenerative medicine strategies to skin repair

Cutaneous ulcers around the ankle, secondary to trauma, vascular insufficiency or diabetes [20,21] are injuries that require special attention mainly owing to low vascular supply, a problem that is of great importance in poor subcutaneous tissue areas.

Simplicity of application and affordable price are the main reasons by which GFs have been widely applied for treatment of different injuries in orthopaedics but also in cardiovascular, plastic surgery and dentistry [22,23]. In a body injury, platelets participate in the natural healing process, being responsible for haemostasis and releasing of bioproteins or GFs that are crucial to the wound-healing process [22,24]. Platelet-rich plasma (PRP) can be harvested from patients’ own peripheral blood and after concentration it becomes ready to be administered at the injury site [25].

**Figure 2.**
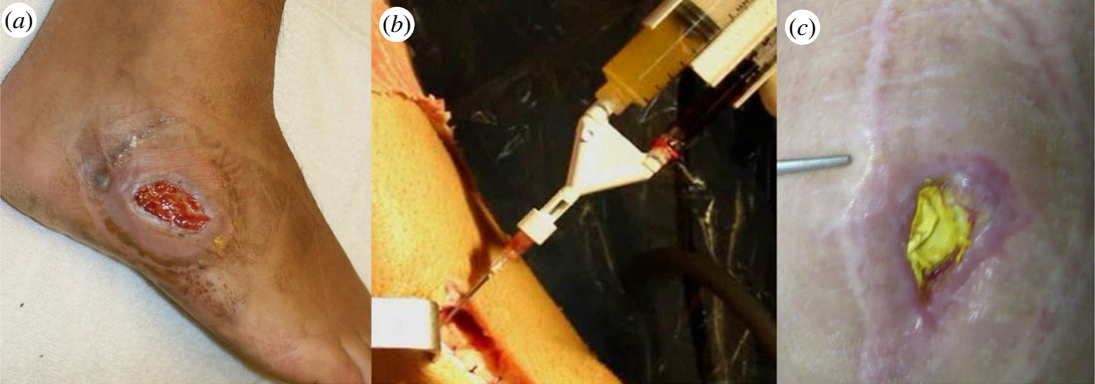
(*a*) Grade 3 ulcer, (*b*) PRP application in wound and (*c*) chronic infected wound protected by collagen membrane with gentamicin sulfate.

Biodegradable biomaterials [21,26] have also been processed as scaffolds and membranes as these systems can act as drug delivery carriers (figure 2), while serving as a three-dimensional template for supporting cell proliferation and repair at the damaged site.

Bone morphogenetic proteins (BMPs) are members of the human transforming growth factor-β (TGF-β) superfamily and similarly to PRP have been demonstrated to have many therapeutic possibilities [27,28]. However, BMPs still present a considerably higher cost as compared with PRP. The biological mechanism of action for BMPs has been demonstrated by Urist [29]. BMP-2 and BMP-7 belong to TGF-β superfamily, and BMP-1 is considered a metalloprotease. It is undeniable the importance of these GFs in the field of tissue engineering, owing to their effect in regeneration of body tissues, specially bone and cartilage. More than 15 BMPs have been described, and their specific characteristics and mechanism of action are under investigation [28].

Tissue-engineered skin with allogeneic dermal fibroblasts and epidermal keratinocytes [21] has been successfully used in chronic wounds that fail to heal with standard wound care. Allogeneic dermal products seem to have the necessary cytokines for wound healing, presenting not only superior effective rate, but also reduced time of treatment. Yamada *et al*. [30] proposed the use of a bilayered hyaluronan/atelocollagen sponge seeded with fibroblasts for wound-healing (e.g. leg, ankle or foot ulcers) applications. That work has shown the beneficial effect of using cell-seeded scaffolds when treating ulcers as it improved wound healing.

TERM approach using acellular dermal graft has also been described [31]. This technique allies tissue-engineered matrices to the cells and GFs present in the human recipient following transplantation. Brigido [32] reported a clinical trial which demonstrated that Graftjacket tissue matrix showed a statistically significant higher percentage of wound healing with respect to wound, and it is more effective than sharp debridement in this small case-control trial. The disadvantage of allogeneic dermal products as compared to the acellular graft is that they require multiple applications and can only be applied to the treatment of superficial full thickness ulcers.

Another relevant issue is related to the treatment of infection in this area. Using TERM technologies such as nanotechnology [17,20,33] (e.g. micro- and nanoparticles or nanospheres developed as systems to deliver drugs in a controlled manner), it is possible to increase simultaneously the delivery of antibiotics at the damaged site and promote tissue repair [13].

#### 1.1.2. Applications of tissue engineering and regenerative medicine strategies to tendon repair

**Figure 3.**
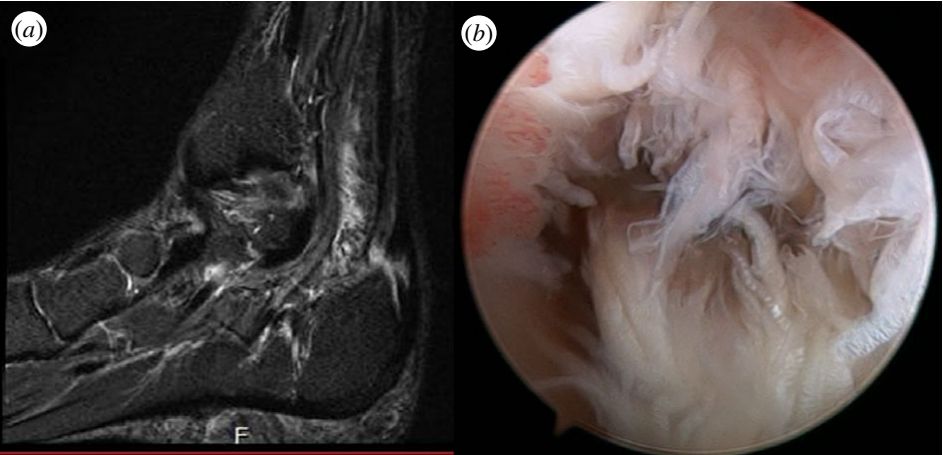
(*a*) Achilles tendon defect partial rupture identified in T2 MRI (arrow) and (*b*) endoscopic view of the defect.

Another relevant group of injuries located in the ankle region is the tendon lesions. Most tendons have the ability to heal after injury, but the newly formed tissue is functionally different from normal tendon. Achilles tendon pathologies (in their several classifications) [34] have high impact in both high-level athletes and the general population. Figure 3 shows a magnetic resonance image (MRI) of a typical Achilles tendon partial rupture. Tendon acute tears treatments are managed by direct suturing techniques [35,36], and the most common form of healing is scar formation. Poor tissue vascularization explains the slow healing rate and the observed scar tissue in the repaired tendon. The latter can affect tissue functioning as scar tissue results in adhesion formation, which disrupts tendons. Therefore, it represents a higher risk of further damage [37–39]. All these facts contribute to distorted motion and consequently reduced life quality [40]. In the last few years, several TERM approaches have been investigated with the promise of a more successful outcome for patients, where acute tendon pathology and chronic tendon ruptures have been diagnosed [41–43]. This can be achieved by means of both inhibiting degeneration process [44–47] and helping to relieve pain [48].

Several GFs have been found to be useful in tendon wound healing [40], like TGF-β [44], BMP, fibroblast growth factor (FGF) [49] and insulin-like growth factor (IGF) [50]. All aforementioned approaches using GFs proved to accelerate the wound-healing process and strength of the repair. However, depending on the concentration, half-time and applied technique, it can also promote undesired fibrosis, with excessive disordered collagen deposition, i.e. the structural properties are improved, but not the tissue functioning [44].

Several studies have reported that PRP has a positive effect on proliferation and metabolism of human tenocytes, and thus enhances tendon repair [22,51]. Meanwhile, the main problem might be the standardization of the methods used in the clinical setting, and concentration of platelets and GFs to be used. One of the most challenging goals is related to the need for establishing the optimal concentration, half-life and local of injection and avoiding clearance of the PRP from lesion sites [48].

**Figure 4.**
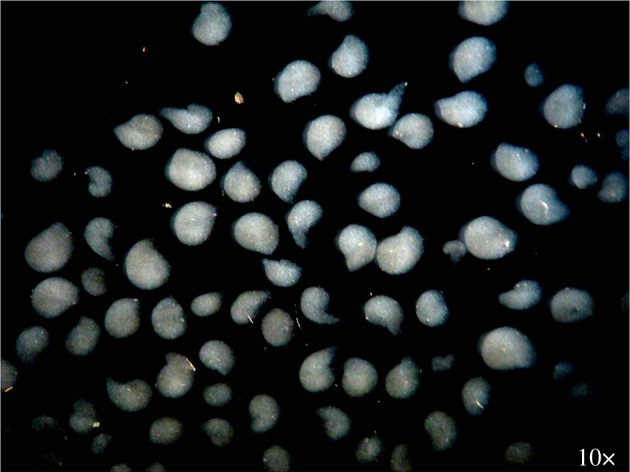
Photograph of the gellan gum microparticles obtained by precipitation in a phosphate buffered saline (pH 7.4) solution and possessing a size between 500 and 2000 µm.

Tenocytes present low mitotic rate, which obviously influences any therapeutic approach. Particularly, in an attempt to reverse/decelerate the degenerative process, controlled drug delivery systems, such as micro- or nanoparticle proteins or polymer-based systems [52,53], have been tried. Figure 4 illustrates gellan gum microparticles obtained by precipitation in a phosphate buffer saline solution*.* Nanotechnology-based approaches are promising when it is envisioned to stabilize and to achieve a controlled release of a given therapeutic agent at the defect site.

Several authors have proposed both acellular and cellular silk fibroin-based scaffolds for ligament/tendon tissue engineering with promising results, *in vitro* and *in vivo* [54,55]. TERM approaches using a ligament/tendon with similar mechanical and functional characteristics as the native tissue can prevent several complications associated with the traditional methods. Scaffolds can be combined with stem cells [15,49,56] or GF [22,24,49,51,57] in a *in vivo* approach (to permit the self-regeneration of small tissue lesions) or used alone [58–60] in an *ex vivo* approach, designed to produce functional tissue that can be implanted in the body. The ideal scaffold for tendon engineering must retain the basic structure of the tendon, mimic native extracellular matrix (ECM) and competence for cell seeding [61]. Reports on the use of several scaffolds (e.g. silk fibroin [54], collagen [45,58], chitosan-based [53] or poly(ethylene glycol) diacrylate hydrogel [62]) combined with adult mesenchymal stem cells (MSCs) demonstrated that differentiation of MSCs into tenocyte-like cells can occur in response to chemical factors, including BMPs, TGF-β and FGF [46,49].

#### 1.1.3. Applications of tissue engineering and regenerative medicine strategies to bone repair

Bone defects and bone reconstruction are, probably, two of the most important issues in a TERM perspective, with several proposals advanced over the years [7,29,53,62,63]. Some injuries in anatomic areas such as distal tibia, talus or calcaneus, given their difficult irrigation and scarce soft tissue protection, usually are difficult to consolidate. This is a particularly critical problem in patients with a clinical history of multiple surgical interventions [33].

Bone grafts can cover the basic requirements for bone repair as they combine a scaffold, GFs and cells with osteogenic potential. Yet, the use of bone grafts is associated with several complications, i.e. non-unions [64], incomplete filling of the defect and late graft fracture [63]. Furthermore, harvesting of autologous bone often results in donor site morbidity, which may vary with the location site and the applied technique [65].

Some technologies combining the use of GFs (namely BMPs) [7,28,29], cells [16] and/or scaffolds [66,67], adapted or not to a surgical intervention have achieved promising results in cases where several previous surgeries have failed systematically [3,33,68].

BMPs, specifically BMP-2, BMP-4 and BMP-7, have been known for over a decade for inducing osteogenic cell differentiation *in vitro* and *in vivo* [68]. The value of recombinant human BMP-2 (rhBMP-2) has been evaluated in a prospective study for treating open tibial shaft fractures [69]. A significant reduction of a secondary intervention was observed in the rhBMP-2 group as compared with the standard care group, suggesting that the use of GFs could accelerate healing of fractures and soft tissue, reduce hardware failure, and thus re-operation owing to delayed healing/non-union. Still, there are only few available GFs for clinical use in bone regeneration besides BMP-2, BMP-7 and growth and differentiation factor-5 (GDF-5) [70]. Recently, Kleinschmidt *et al.* [70] reported that the use of a mutant GDF-5 (obtained by introducing BMP-2 residues into GDF-5) demonstrated enhanced osteogenesis and long bone formation capacity [70]. When the use of GFs alone is not recommended, as in the treatment of large bone defects, stem cells and scaffolds are a very promising alternative to standard procedure. Stem cell-based TERM strategies require three main steps: (i) cells are harvested, isolated and expanded, (ii) scaffolds are seeded with the induced cells, and (iii) cell-seeded scaffolds are re-implanted *in vivo* [68]. The aim of TERM is the substitution of the missing tissue with the *ex vivo* tissue-engineered construct. There are several reports [71,72] on the application of different scaffolds combined with stem cells (mostly MSCs derived from bone marrow or adipose tissue). These have shown favourable autogenous bone grafting and no donor site morbidity [68]. Scaffold choice is still under investigation in order to be standardized. Biodegradable synthetic polyesters [73], calcium phosphate ceramics [74,75] and chitosan–alginate [76] are some of the scaffolds that have proved to have significant value in the treatment of bone defects.

Cancedda *et al.* [63] have provided relevant information and new insights on the importance of scaffold architecture towards enhancing de novo bone formation within scaffolds *in vivo*.

Kokemueller *et al.* [77] have been also investigating the vascularization of seeded scaffolds required for clinical application in reconstructive cranio-maxillofacial surgery. The authors reported that prefabrication of vascularized bioartificial bone grafts *in vivo* might be an alternative to *in vitro* tissue engineering techniques as it presented minimal donor site morbidity and no shape or volume limitations.

More recently, Nagata *et al.* [78] reported the use of cultured autogenous periosteal cells (CAPCs) in alveolar bone regeneration. CAPCs were mixed with particulate autogenous bone and PRP and grafted into the injury sites. Results have shown that CAPC grafting enhances recruitment of both osteoblasts and osteoclasts, accompanied by angiogenesis and leading to satisfactory bone regeneration.

**Figure 5.**
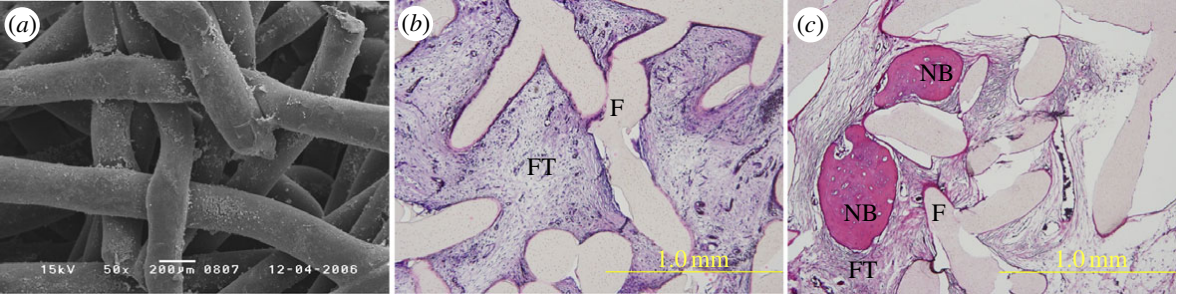
(*a*) Scanning electron microscopy image of MSCs seeded onto SPCL scaffolds and maintained in a standard osteogenic culture medium, after 14 days of culturing. Microscopy images of histological sections (haematoxylin and eosin staining) of (*b*) SPCL scaffold controls and (*c*) MSCs/SPCL construct explants after four weeks of implantation (Fischer rats subcutaneous model). Newly bone formed (NB), SPCL fibres (F) and fibrous tissue (FT).

Oliveira *et al.* [79] proposed the combination of nanotechnology tools and tissue engineering approaches for pre-programming the fate of bone marrow stromal cells (BMSCs) towards promoting superior de novo bone formation. The authors have shown that BMSCs cultured *in vitro* (figure 5) with a dendron-like nanoparticles system that delivers dexamethasone intracellularly, seeded onto starch–polycaprolactone (SPCL) scaffolds (figure 5*a*) and implanted subcutaneously were able to differentiate and produce new bone, *in vivo* (figure 5*c*). That work clearly evidenced the advantages of using intracellular tools, for example the dendronized nanoparticles, for tuning stem cells *in vivo*.

## 2. Osteochondral ankle lesions

Osteochondral defects (OCDs) and osteoarthritis in lower limb have a relevant socio-economic impact with significant therapeutic investments and absence from work-related costs [80,81]. OCDs are defined as lesions of any origin that involve the articular surface and/or subchondral region, thus affecting cartilage, bone or both [81]. Suggested causes of ankle OCDs include local avascular necrosis, systemic vasculopathies, acute trauma, chronic microtrauma, endocrine or metabolic factors, degenerative joint disease and genetic predisposition [82].

Asymptomatic OCD patients can be treated non-operatively, with rest, ice application and immobilization or temporarily reduced weight bearing, even though this management has shown relatively high rates of failure [83].

Symptomatic patients with OCDs should be treated surgically. The main aim is to promote re-vascularization of the bone defect [84–86]. This goal is achieved applying three principles [87]: (i) debridement and bone marrow stimulation (e.g. microfracture, drilling and abrasion arthroplasty), (ii) securing a lesion to the talar dome (e.g. fragment fixation, retrograde drilling and bone grafting), and (iii) development or replacement of hyaline cartilage (e.g. autologous chondrocyte implantation (ACI), osteochondral autograft transplantation (OAT), mosaicplasty and allografts) [88].

Articular hyaline cartilage is avascular and it has poor regenerative capability [89,90]. When repair involves the formation of fibrous cartilage, the newly formed tissue will lack favourable biomechanical properties and it can fail [90]. Therefore, the damaged tissue should be replaced with a tissue that best resembles the native hyaline cartilage [81,88]. For this reason, significant economic and scientific investments have been made on TERM applications in the treatment and prevention of cartilage defects and joint degradation [33]. Minimally invasive methods that can facilitate their use have also attracted much attention [81,88,91,92]. Besides, prolotherapy and arthroscopic/endoscopic procedures have a lower risk of complications. These procedures facilitate and decrease rehabilitation time, thus they help fight absence from work and promote return to athletic activity [88,92]. TERM strategies have been developed or adapted to promote this kind of application/delivery [81,88,92].

### 2.1. Applications of tissue engineering and regenerative medicine strategies to ankle osteochondral lesions repair

#### 2.1.1. Applications of isolated growth factors

Debridement and bone marrow stimulation have been used as surgical approaches for partially destroying the calcified zone that is often present in OCDs and to create multiple openings into the subchondral bone [87,89]. As a consequence of these interventions, intra-osseous blood vessels are disrupted, and the release of GFs can lead to the formation of a fibrin clot and fibro-cartilaginous tissue formation. These approaches have proven to be one of the most effective treatments for OCDs of the talus, especially in a small lesion (less than 6 mm), with minimal subchondral bone involvement [81,87].

Based on this surgical treatment, the use of isolated GFs in the treatment of symptomatic OCDs has undergone a huge expansion over the last few years [33]. In the body's natural response to injury, a complex healing process is initiated. Platelets participate in this process, as they are responsible for stopping bleeding and for haemostasis [22]. Once they are activated by mediators at the site of injury, they undertake degranulation, releasing GFs that will help the wound-healing process. Examples of these GFs are TGF-β, IGF-1 and IGF-2, FGF, all of which have been shown in experimental settings to promote healing and the formation of the new tissue [8].

The short half-life of these proteins, the difficulty in keeping them within the area of the defect and the low mitotic rate of chondrocytes, among several other issues, make it hard or even impossible to predict, from a theoretical perspective, the complete repair of a chondral defect or OCD using this approach [28]. Moreover, results available in the literature are controversial, with some series reporting significant clinical or symptomatic improvement [17,24], while other studies conclude that there is not enough evidence to support their use with this objective [8]. Two recent reports have used TGF-β, IGF-1 and BMP-2 associated with scaffolds and have reported promising results for the repair of OCDs and cartilages [93,94]. Although the anabolic effect of these GFs cannot be questioned, as has been demonstrated and confirmed *in vitro* and *in vivo* [95,96], the original tissue replacement for fifibrous tissue is commonly observed in the neo-surface of the OCDs [2,94].

It is consistently recognized that most of the published studies have a low methodological quality in this matter, i.e. besides the absence of uniform criteria in outcome assessment, most of them also do not consider or not specify the different GFs applied, their quantities, isolation methods, simultaneous presence or absence of other proteins (e.g. metalloproteases) or cells (e.g. leucocytes) [22,25,97]. It becomes obvious that the improper early use of a promising technique will lead to obstacles in its correct improvement which creates higher resistance to its future application. However, tissue repair and homeostasis depend on a multitude of factors (the TERM triad) and should not be lightly simplified this way. Research must still progress considerably to gain deeper knowledge on the GFs application and their effects on different tissues and clinical situations. Thus, GFs are probably not expected to be a panacea, being able to solve all our problems independently of the way they are produced, stabilized and administered to the patient.

Besides the previously stated, one cannot ignore the analgesic effects which simple platelet-derived GF methods have shown in several clinical trials [8,22], particularly among high-level athletes.

#### 2.1.2. Applications of isolated cells

MSCs have demonstrated their high potential for clinical use as therapeutic agents with several possible RM applications including orthopaedics and percutaneous (injectable) techniques [98].

The rehabilitation of injured/degraded cartilage through the degenerative process leading to osteoarthritis remains the main challenge that clinicians and researchers have been facing. Several researchers have tested the use of MSCs instead of chondrocytes in the attempt to repair cartilage defects and defend joint homeostasis [71,99,100].

MSCs have the capacity to modulate the immune response of the individual and positively influence the microenvironment of pluripotent cells already present in native injured tissue. Through direct cell-to-cell interactions or by secreting a number of different proteins, MSCs can promote the endogenous regenerative mechanisms still present in an arthritic joint [101].

Gene therapy with modified MSCs might increase this therapeutic field in the near future [68,96,101]. Besides their isolated application, MSCs’ chondrogenic differentiation can be induced at the target tissue or in combination with an adequate support scaffold [99]. This may obviate the limited lifespan of chondrocytes that is an obstacle in the treatment of large OCDs [102].

Another therapeutic possibility makes use of cultured chondrocytes, which are expanded and finally implanted at the defect site [103]. ACI is an alternative to OAT and it involves harvesting a small amount of cartilage for chondrocyte isolation and culturing (*in vitro*), usually from a knee ipsilateral to the ankle injury [87,88,103]. Cell-based techniques have gained relevance in OCDs because, unlike bone-marrow-stimulation methods, where fibrocartilage fills the defect, cells can potentially induce regeneration and produce a ‘hyaline-like cartilage’ [104]. Nevertheless, a recent study [105] has shown that chondrocytes from the injured zone in the ankle have poorer regenerative capacities as compared with normal tissue, stating some reservations to their use in the therapeutic field. Thus, it seems that the source for harvesting cells should be a normal, healthy tissue, requiring one additional surgical procedure and limited associated morbidity.

On the other hand, the differentiated cells are sensitive and can present biochemical changes or diminished viability during the processes of harvesting, culturing, expansion or re-implantation in the defect zone [6].

The potential of ACI in the treatment of OCDs has been the source of great enthusiasm since the study performed by Brittberg *et al*. [103]. After 3 years of follow-up, the transplants restored considerable knee function in 14 of the 16 patients with femoral defects. The treatment resulted in the formation of new cartilage that was similar to normal cartilage in that it had an abundance of type II collagen and metachromatically stained matrix, similar as in original cartilage.

Still, despite several successes reported by the followers of this technique [106], up to now there is no evidence-based medicine to support their use, with no proven cost-effective advantages as compared to ‘classic’ treatment options such as microfractures or osteochondral grafting techniques (OAT, mosaicplasty) [107–111].

Some advocate specific conditions for its use, for example a defect area more than 4 cm^2^ (factor predictor of a better outcome with ACI), reinstating the existence of specific injury and individual's conditions which might play a determinant role in outcome [112]. As aforementioned, gene therapy can enhance the clinical application of differentiated cells as stated by Orth *et al.* [113]. That study demonstrated that chondrocytes modified for higher co-expression of IGF-1 and FGF-2 hold an increased chondrogenic capacity *in vivo*.

#### 2.1.3. Applications of biomaterials

Hyaline cartilage serves as a low-friction surface with high wear resistance for weight-bearing joints. Unfortunately, it possesses an avascular and alymphatic profile which limits its autonomous regenerative capacity. The application of differentiated cells in the clinic presents additional problems such as cells' tendency towards losing their differentiated phenotype in a two-dimensional culture (e.g. chondrocytes) and to differentiate towards a fibroblast-like phenotype [114]. To overcome this problem in the treatment of cartilage lesions, different scaffolds have been developed for supporting cell adhesion, proliferation and maintenance of phenotype in an effective manner [4,115].

Among the several scaffolds proposed in an attempt to better fulfil the requirements of cartilage regeneration process, there are substantial differences regarding the materials chosen and their physical forms (i.e. fibers, meshes and gels). Solid scaffolds provide a substrate on which cells can adhere, whereas gel scaffolds physically entrap the cells [116]. The biomaterials used can be classified as synthetic or natural. Synthetic matrices present mechanical properties and degradation rates more easily tuned as compared with that of natural polymers, but some biocompatibility concerns might be raised owing to their degradation products and potential effect on native tissue and implanted cells. However, innovations in chemistry and materials science have been improving their biocompatibility [116]. Among the natural and synthetic materials that have been investigated (e.g. gellan gum, alginate, silk fibroin, chitosan, hydroxyapatite, collagen, hyaluronic acid (HA), polyglycolic acid and polylactic acid) [117], few have been used in ankle lesions, probably due to the lack of studies in the field of ankle tissue regeneration. Table 1 [5,53,93,118–127,129,130,132–140,142–145,147–151] summarizes the most important reports on polymers, ceramics and composites that have been used as scaffolds for osteochondral tissue regeneration.

**Table 1.** Biomaterials used in the preparation of scaffolds for osteochondral tissue regeneration.

	repeating unit	properties	examples of proposed applications
natural polymers			
collagen	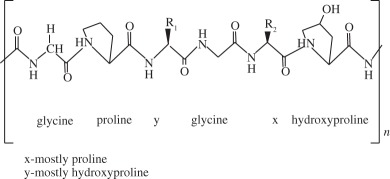	it is the most abundant protein in the body. It possesses high mechanical strength, good biocompatibility and low antigenicity, which make it suitable for tissue engineering. Combinations of other materials are also described, as well as GFs or cell implantation	atelocollagen gel was reported to be successfully used on OCDs on talar dome [118] collagen bioscaffold seeded with autologous chondrocyte for the treatment of OCDs in rabbit knee [119] collagen biphasic-based scaffolds were used in OCDs of the goat and compared to PLGA. Both provide indications of satisfactory development of a structural repair [120]
silk fibroin	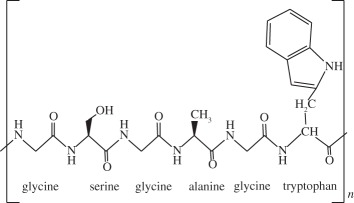	it contains a highly repetitive primary sequence that leads to a high content of β-sheets, responsible for the good mechanical properties of silk fibres. It has been shown to be a biocompatible material that allows good cell attachment, providing an adequate three-dimensional porous structure and the necessary mechanical support for bone and cartilage tissue generation	porous silk scaffolds, bioreactors and BMSCs were used to engineer cartilage- or bone-like tissue constructs [121] silk fibroin scaffolds were reported to be suitable for use in meniscus and cartilage tissue-engineered scaffolding [5] rabbit BMSC/silk fibroin scaffold-based co-culture approach was used to generate tissue-engineered osteochondral grafts [122]
alginate	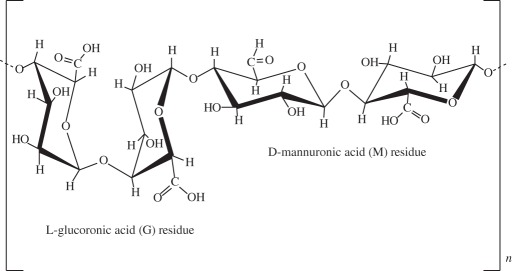	it is non-toxic, biocompatible and biodegradable natural polymer that is widely applied in drug and cell delivery systems. Hydrogel formation can be obtained by interactions of anionic alginates with multivalent inorganic cations by simple ionotropic gelation method. Hydrophilic polymeric network of three-dimensional cross-linked structures of hydrogels absorbs substantial amount of water or biological fluids	alginate droplets were gelated to form a highly organized scaffold and the feasibility of the use of this scaffold in cartilage tissue engineering was demonstrated [123] alginate-based bilayered scaffolds loaded with GFs on rabbit knee [93]
chitosan	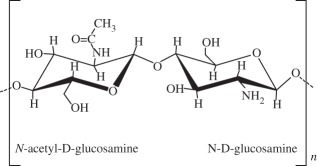	it is a derivative of chitin and partially de-acetylated. Structurally, chitosan is a linear polysaccharide that shares some characteristics with various glycosaminoglycans and hyaluronic acid present in articular cartilage, composed of glucosamine and *N*-acetyl glucosamine. Some important properties are its biocompatibility, biodegradability, antibacterial activity, mucoadhesivity and wound-healing ability	development of novel hydroxyapatite/chitosan bilayered scaffold that shows potential for being used in TE of OCDs [124] appropriate chitosan properties were evaluated for an *in vivo* osteochondral tissue regeneration on rabbit knee [125]
hyaluronic acid	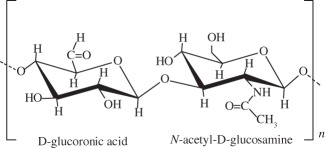	one of the most important components of the ECM. Is soluble in water and can form hydrogels by covalent and photo-cross-linking, esterification and annealing. It is enzymatically degraded by hyaluronidase. The degradation products of hyaluronan, the oligosaccharides and very low-molecular-weight hyaluronan exhibit pro-angiogenic properties and can induce inflammatory responses in macrophages and dendritic cells in injured tissues	*in situ* photo-cross-linkable hyaluronan was developed and evaluated as a scaffold for articular cartilage repair *in vitro* [126] MSCs were seeded in a hyaluronan scaffold for repair of an OCD in rabbit knee [127]
gellan gum	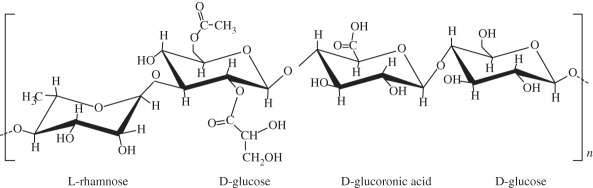	it forms thermoreversible gels possessing mechanical properties varying from soft to elastic. Presents no toxicity and it could be used in a non-invasive manner. Similar structure to native cartilage glycosaminoglycans	gellan gum adequately supported the growth and ECM deposition of human articular chondrocytes implanted subcutaneously in nude mice [128] successful encapsulation of human nasal chondrocytes on gellan gum [129] gellan gum hydrogels seeded with autologous cells proved to be a promising approach in treatment of cartilage defects in rabbit knee [129,130]
synthetic polymers			
poly(ethylene glycol) derivatives	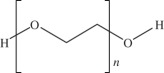	synthetic hydrogels are water-swollen polymeric networks, usually consisting of cross-linked hydrophilic polymers that can swell, but do not dissolve in water. This ability to swell under biological conditions makes them an ideal class of materials for biomedical applications, such as drug delivery systems and tissue engineering scaffolds for cell encapsulation. Hydrogels possess a three-dimensional network structure, cross-linked together either physically or chemically. This insoluble cross-linked structure allows effective immobilization and release of active agents and biomolecules or even cells. Generally exhibit good biocompatibility and high permeability to gases, nutrients and other water-soluble metabolites, making them attractive scaffolds	poly(ethylene glycol)-based hydrogels used in osteochondral knee defect in rats [132] oligo[poly(ethylene glycol) fumarate] hydrogel alone or loaded with BMSCs to endorse fully repair of OCDs on porcine model [133,134]
PLGA	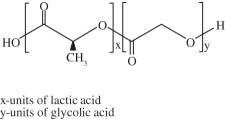	biodegradable and biocompatible and having mechanical strength, suitable for cartilage repair. It can be tuned with different pore size along the scaffold and combined with other polymers, for example polyurethane. It is suitable for seeding with BMSCs and GFs	biphasic cylindrical porous plug of PGLA with β-tricalcium phosphate was used to repair articular cartilage in porcine model [135] PLGA scaffold was implanted into OCDs on femoral trochlea of rabbits [136] bilayered porous scaffolds seeded with BMSCs for regeneration of OCDs on rabbit knee [137] PLGA-based bilayered scaffolds loaded with GFs on rabbit knee [138]
poly(l-lactic acid) (PLLA)	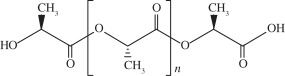	biodegradable polyester that exhibits mechanical properties suitable for bone tissue regeneration. It degrades by hydrolytic scission of its ester bonds, yielding the physiologic molecule lactic acid. As a biodegradable material, it is suitable for tissue engineering, owing to the fact that the newly formed tissue can invade the space while the material degrades	PLLA-based scaffold incorporated with GFs was used to repair articular cartilage defect in a rabbit model [139] PLLA/hydroxyapatite nanocomposites induced differentiation of hMSCs in a chondrocyte-like phenotype with generation of a proteoglycan-based matrix [140] optimization of the mineralization process on a PLLA macroporous scaffold on OCDs performed in the medial femoral condyle of healthy sheep [141]
polycaprolactone (PCL)	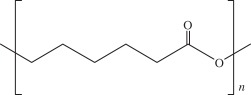	it is one of the most widely used biodegradable polyesters for medical application owing to its slow biodegradability, biocompatibility, mechanical properties and structural flexibility. PCL expresses slow degradation kinematics and its degradation products are harmlessly metabolized in the tricarboxylic acid cycle	three-dimensional PCL scaffolds with BMP-2 were applied to investigate the influence of BMP-2 on cartilage matrix and bone matrix production [142] nanostructured porous PCL scaffold was developed to stimulate articular cartilage repair. It improved chondrocytic differentiation to produce more hyaline-like tissue [143]
ceramics	chemical structure	properties	examples of proposed applications
hydroxyapatite	Ca_10_(PO_4_)_6_(OH)_2_	it presents high biocompatibility, but low strength and fracture toughness, which may be a problem in OCD engineering. The osteocondutive properties of hydroxyapatite-based materials can be improved by manipulation of the structural characteristics	implants load with BMSCs have proved to be useful in bone repair of sheep long bones [144] trilayered scaffold with collagen and hydroxyapatite used on osteochondral regeneration in the femoral condyles of the sheep [145,146] composed with zirconia has been proved to be an effective scaffold for cartilage tissue engineering [147]
aragonite	Ca(CO_3_)	it is a biological material very similar to bone, including its three-dimensional structure and pore interconnections that confer osteoconductive ability. Nevertheless, the native material does not regenerate hyaline cartilage	aragonite–hyaluronate bi-phasic scaffold showed cartilage regenerative potential in a goat model [148]
tricalcium phosphate	Ca_3_(PO_4_)_2_	it is a calcium salt of phosphoric acid, widely used as a synthetic alternative owing to their chemical similarity to the mineral part of the bone. Presents a high osteoconductivity and a cell-mediated resorption. Calcium and phosphate ions released during the resorption can be used to mineralize new bone in the bone remodelling process. It may be used alone or in combination with a biodegradable and resorbable polymer, for example polyglycolic acid	tricalcium phosphate-based scaffold loaded with GFs was reported to induce chondrogenic differentiation, tissue formation and differentiation in a mini-pig model [149] microporous three-dimensional calcium phosphate was seeded with autologous chondrocytes and implanted in femoral condyle of ovine knees [150,151]

Biomaterials including ceramics and polymers, such as aragonite [148], silk fibroin [5,121] or tricalcium phosphate [149–151], are some of the most promising materials for OCD regeneration, alone or alternatively blended with other materials.

**Figure 6.**
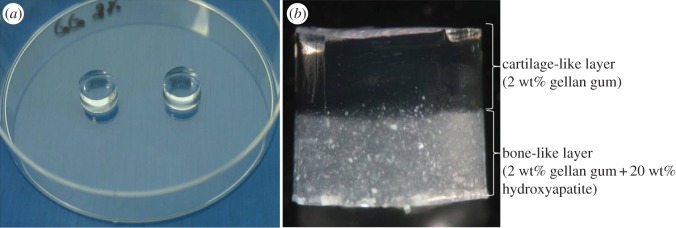
Photographs of gellan gum hydrogels: (*a*) single and (*b*) bilayered.

The application of an injectable biomaterial with bioadhesive properties, for example gellan gum (figure 6*a*), for regeneration of cartilage has been proposed for the first time by Oliveira *et al.* [129]. The gellan gum hydrogel was shown to efficiently sustain the delivery and growth of human articular chondrocytes and support the deposition of a hyaline-like ECM [128], leading to the formation of a functional cartilage. The use of biocompatible gellan gum-based hydrogels (e.g. methacrylated gellan gum, GG-MA) is also justified due to their many advantages such as improved biostability, tuneable degradability, mechanical properties and bioadhesiveness [52,130,131]. The versatility of the injectable gellan gum hydrogels and functionalized derivatives allowed the development of ionic- and photo-cross-linked GG-MA hydrogels, with improved mechanical properties for *in situ* gelation, within seconds to a few minutes [152,153]. Besides being able to serve as carriers of GFs/drugs and/or cells and promote ECM production, in another study [154], GG-MA hydrogels have been shown to possibly enable the control of the neovascularization process. In other words, one can use two different forms of gellan gum-based hydrogels to transport different cells: (i) in a given zone, facilitate vascular ingrowth (e.g. area to integrate in subchondral bone in a grade IV injury according to International Cartilage Repair Society) and (ii) in another area, prevent neovascularization and re-innervation by the presence of the hydrogel itself while it can also transport chondrocytes to the region that will replace hyaline cartilage [154]. That important work brings new insights to mimicking more precisely the native properties of tissue, because different tissues require neovascularization for regeneration, as in others vascularization and re-innervation is associated with pain and degeneration [155]. In fact, one of the goals of TERM is, precisely, to maintain the human characteristics of the natural tissue and so the knowledge of physiology of the original tissue is crucial.

Another biomaterial that has been tested, including in talar dome resurfacing, is collagen in its many presentations [66]. Besides its biocompatibility and positive results for the management of painful post-traumatic of the ankle joint, the biomechanical properties and stability remains an issue in several of its applications [66,118].

Hydrogel systems have been developed to obtain optimal nutrient diffusion [40,49], connectivity with host matrix, adequate biodegradability, solubility and mechanical properties to facilitate the production and organization of the matrix [14]. Several improvements have been achieved with several former systems, but the ‘ideal’ one remains to be established [156]. One of the most studied hydrogels is based in HA. The use of HA as adjuvant of microfractures surgical treatment (i.e. bone-marrow-stimulation techniques) seems to improve the results of microfractures alone, taking advantages of HA's rheological properties [157].

**Figure 7.**
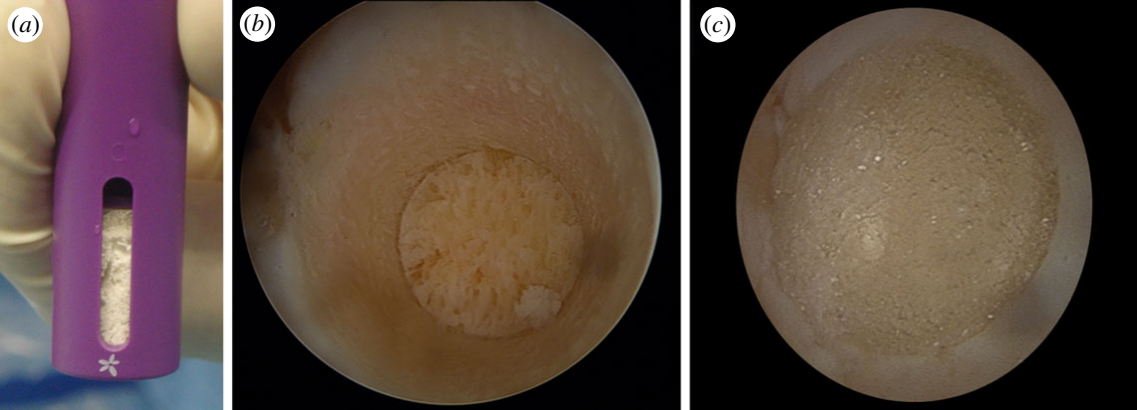
(*a*) Photograph of TruFit PLGA-based scaffold delivery device, (*b*) defect zone prepared to receive the plug and (*c*) arthroscopically implanted device to resurface the defect preserving joint congruency.

Since a treatment that focuses exclusively on articular cartilage is likely to fail [90], it has been suggested that treatment strategies should be designed with the entire osteochondral unit (articular cartilage and subchondral bone) [90]. Therefore, bilayered porous scaffolds with poly(lactide-*co*-glycolic) (PLGA) seeded with BMSCs [137] or with GFs [138] were reported to simultaneously regenerate cartilage and subchondral bone of rabbit knee. Porous PLGA–calcium sulfate biopolymer (TruFit by Smith and Nephew, London, UK) is one of the most popular commercially available devices (probably the most clinically tested) [135,136], and it has been applied from mono- to bilayered presentations (figure 7). Jiang *et al.* [135] observed bone formation in the osseous phase, with evident subchondral remodelling, as well as normal hyaline cartilage, in a mini-pig model, when cell suspension (composed of the harvested autogenous cartilage) was injected into the chondral phase of the PLGA scaffold. More recently, this device is also available in a shape adapted to the anteromedial talar corner. However, there is still little evidence-based medical data supporting its use in either acellular or cellular strategies, besides the existence of some concerns with polyglycolic acid biocompatibility [90,158].

In the field of ceramic polymers, hydroxyapatite is one of the most used implant materials for medical applications owing to its high biocompatibility [144]. It seems to be the most appropriate ceramic material for cartilage tissue engineering. However, owing to low strength and fracture toughness of the material, new approaches have been reported [147] in order to achieve a scaffold with the most suitable properties for cartilage tissue engineering. Sotoudeh *et al.* [147] reported that a composite of zirconia and hydroxyapatite would be an effective scaffold for cartilage regeneration.

The use of bilayered scaffolds (figure 6*b*) that combine different materials in the same implant constitutes a natural evolution in OCD treatment, in an attempt to combine favourable properties to both bone integration and cartilage repair [124,159]. In fact, it has been shown that the hydroxyapatite layer permits adhesion and proliferation of MSCs and osteogenic differentiation *in vitro* [124], while facilitating new bone formation *in vivo* [72]. By its turn, the cartilage-like layer is also able to support the adhesion of MSCs and can promote chondrogenesis, *in vitro*.

Another important commercially available product is MaioRegen (Fin-Ceramica SpA, Faenza, Italy) [146,160], which is a trilayered scaffold for treatment of OCDs. The deepest layer is composed of hydroxyapatite, the intermediate layer is a mixture of type I collagen and hydroxyapatite and the superficial layer consists of type I collagen only. In a previous study performed *in vitro* and *in vivo*, Kon *et al.* [145] obtained similar results when the scaffold was loaded with autologous chondrocytes or when it was used alone. The ability of the scaffold to induce OCD repair without the seeding of autologous cells makes it very attractive [146]. Comparative studies with OAT, ACI and bone-marrow-stimulation techniques are needed to establish the clinical outcome of this procedure.

#### 2.1.4. Applications of advanced tissue engineering and regenerative medicine strategies

The requirement for full OCD repair has been approached considering the heterogeneity of different tissues, different components and layers (including subchondral bone plate and different hyaline cartilage layers). This is also part of the underlying principle for OAT. Although some attempts have been made to overcome one of the most relevant problem of OAT [107], relevant morbidity related to donor zone in knee-to-ankle transplantation has been demonstrated [110,161]. Furthermore, other problems persist with these techniques including graft's source, achievement of joint congruence and interface between graft plugs and between grafts and native cartilage. It is generally accepted that the use of a lower number of plugs is a predictor of a better mid- to long-term outcome [107].

Table 2 [162–165] summarizes the most important clinical studies related to TERM strategies for treatment of ankle lesions. Those studies have tested two main biomaterials, i.e. collagen and hyaluronan-based scaffolds/membranes, with matrix-induced ACI (MACI, Verigen, Leverkusen, Germany) being the most used approach. This technique can be considered as an evolution of conventional ACI and it makes use of processed cells that are harvested and isolated from the patient and expanded *in vitro*. Once grown, the chondrocytes are seeded between layers of a bilayered collagen scaffold in the operating room, prior to implantation of cell–scaffold construct into the defect area.

**Table 2.** Clinical studies on TE of cartilage/OCD of the ankle.

references	biomaterial/treatment approach	defect area/follow-up	procedure	outcome
Giannini *et al.* [162]	Hyalograft C scaffold seeded with human autologous chondrocytes	ankle/12 and 36 months	patients (*n* = 46) with a mean age of 31.4 years, post-traumatic talar dome lesions. First procedure: ankle arthroscopy to harvest cartilage. Chondrocytes were cultured on Hyalograft C scaffold. In the second step, the construct was arthroscopically implanted into the lesion site. Patients were evaluated by AOFAS score pre-operatively and at 12 and 36 months post-surgery	the mean pre-operative AOFAS score was 57.2 ± 14.3. After 12 and 36 months, the scores were 86.8 ± 13.4 and 89.5 ± 13.4, respectively. Clinical results were significantly related to the age of patients and to previous operations for cartilage repair. Histological stainings have revealed that hyaline-like cartilage was formed
Giannini *et al.* [163]	collagen powder/hyaluronan membrane loaded with concentrated BMDCs	ankle/6, 12, 18 and 24 months	patients (*n* = 23) used collagen/MSCs, and 25 patients used hyaluronan/MSCs for the treatment. Porcine collagen powder (Spongostan Powder) and hyaluronic acid membrane (HYAFF-11) were used. At first, bone marrow was harvested and concentrated. Then, the collagen powder or hyaluronan membrane was mixed with bone marrow and platelet-rich fibrin gel and composites were implanted	for the collagen powder group, the mean AOFAS scores of pre-operation and 24 months post-operation were 62.5 ± 18 and 89.8 ± 9.8, respectively. In the hyaluronic acid group, the scores increased from 66.2 ± 10.5 to 92.8 ± 5.3, 24 months after the surgery. At 2 years follow-up, MRIs showed the restoration of the cartilage layer and subchondral bone of the patients
Giza *et al.* [164]	collagen type I/III bilayered membrane with autologous chondrocytes	ankle/1 and 2 years	patients (*n* = 10) with average age of 40.2 years. The size and location of the defects were analysed by ankle arthroscopy, and cartilage was also harvested from the border or the lesion. Expanded chondrocytes were seeded into the collagen membrane. The joint was exposed with a small anterolateral or anteromedial approach, without malleolar osteotomy. The graft was cut and placed into the defect on top of a layer of fibrin sealant	the AOFAS hindfoot scores increased from 61.2 (pre-operative, ranged from 42 to 76) to 74.7 (1 year post-operative, ranged from 46 to 87) and 73.3 (2 year post-operative, ranged from 42 to 90). At 19 months post-operation, MRIs showed the regeneration of articular cartilage and subchondral bone
Aurich *et al.* [165]	collagen type I scaffold with autologous chondrocytes (MACI)	ankle/mean follow-up 24.5 months	patients (*n* = 18, with a total of 19 defects) with mean age of 29.2. Arthroscopy was used for the evaluation and debridement on the defects, as well as the harvest of cartilage. Cultured chondrocytes were seeded into the collagen membrane and implanted in the defects, with fibrin as the glue. MOCART score, the pain and disability module of the foot function index (FFI), AOFAS score and the core scale of the foot and ankle module of the American Academy of Orthopaedic Surgeons (AAOS) lower limb outcomes assessment instruments were used	according to AOFAS hindfoot score, 64% were rated as excellent and good, whereas 36% were rated fair and poor. The results correlated with the age of the patient and the duration of symptoms, but not with the size of the lesion. Mean MOCART score was 62.4 ± 15.8 points. There was no relation between MOCART score and the clinical outcome

The studies that have been reported demonstrate [119,121,133,151] that combination of scaffolds and autologous cells can enhance the regeneration outcome, using scores adopted either by American Orthopaedic Foot and Ankle Society (AOFAS) or Magnetic Resonance Observation of Cartilage Repair Tissue (MOCART).

Cellular-based techniques, such as ACI and MACI, require a two-stage operative procedure, where initial harvesting of cartilage is followed by culturing and subsequent implantation of the cultured tissue. In fact, this issue has been considered one of the major drawbacks of ACI. This has been the driving force for the search for new treatment methods [166] and development of novel and bioactive scaffolds, which can be easily implanted and fixed, and best mimic the native tissue to be repaired. The use of bilayered tridimensional porous scaffolds enhanced by MSCs requires several years of preclinical research [124]. Still, it remains a trend with high interest and investment from the scientific community. The histological results are available only in animal studies, but are indeed very encouraging [145]. Clinically, they have been applied up to now only in the knee, but they may represent a solution for the repair of deep OCDs even in the ankle [100,146]. The development of the ideal scaffold has been performed in a stepwise manner and is dependent on the knowledge gained in the last few years, in what concerns the biomechanical and biological properties of native tissues [5].

MSCs are emerging as a powerful tool for treatment of cartilage lesions, thanks to their ability to differentiate into various lineages [167]. In particular, the use of concentrated bone marrow instead of chondrocytes, in order to provide MSCs to be seeded onto the scaffold, has been recently introduced in clinical practice as a one-step procedure for the treatment of OCDs.

Giannini *et al.* [163] described their experience with bone-marrow-derived cells (BMDCs) implanted in talar dome focal OCDs. Two types of scaffolds were tested. Both collagen powder and hyaluronic acid membrane showed similar clinical improvement at 2 years in AOFAS score and a good MRI. Recently, the same group [168] compared the clinical outcome in focal osteochondral monolateral talar dome lesions after three different surgical approaches: (i) open fifirst generation ACI, (ii) arthroscopic *Hyalograft C* (Fidia Advanced Biopolymers Laboratories, Padova, Italy) implantation, and (iii) arthroscopic repair by BMDC implantation on a hyaluronic acid membrane. Although similar pattern of improvement was found at 3 years follow-up in all groups regarding collagen type II and proteoglycan expression, BMDCs showed a marked reduction in procedure morbidity and costs, demonstrating it to be a one-step technique able to overcome most of the drawbacks of previous techniques. Nearly complete integration of the regenerated tissue with the surrounding cartilage was demonstrated in 76% of the cases. In addition, histological analysis highlighted the presence of all components of hyaline cartilage in repaired tissue, which showed various degrees of remodelling.

Finally, Battaglia *et al.* [169] confirmed the good results of BMDC transplantation, with 85% of good to excellent clinical outcome, and demonstrated the ability to regenerate hyaline cartilage but not the capability of osteogenesis in OCD repair. In fact, regenerated mature bone was evident only in two cases and in less than 8% of regenerated volume. It must also be kept in mind that the phenotypic preservation of chondrocytes and/or adequate manipulation of MSC differentiation process in different tissues remain as challenging unsolved issues. Chondrocytes are ‘fragile’ cells, exposed to de-differentiation during laboratory manipulation (loss of original phenotype) [59,68,111]. The differentiation of MSCs into chondrocytes is a multi-factorial, complex target which requires, *in vitro*, the contemplation of simulators of biophysical stimulus present in normal tissues—bioreactors [26,135,136,158,170]. Both cell types remain under preclinical investigation and the bench-to-bedside transfer is still an unclosed matter.

The treatment of different focal OCDs by means of using autologous chondrocyte transplantation in tridimensional support scaffolds has been recently attempted [10,108,112,164]. Aiming to enhance this therapeutic strategy, the simultaneous application of GFs has also been evaluated, attempting to favour local environment for short-term integration and promote differentiation [10,11].

A recent study comparing two commercially available methods, (i) *Hyalograft C* (used by arthroscopic application) and (ii) *Chondro-Gide MACI* (open surgery application), concluded that both methods led to positive results, but the method of application influenced short-term results [171]. Arthroscopic application seems to provide faster rehabilitation, despite no significant differences being noted at 2 years follow-up. The reported failure rate was globally 20% highlighting the need for improvement of both techniques. The authors considered results as fair/good and recommended consideration of these techniques when debridement and bone marrow stimulation fail [171].

Gene therapy can provide some new answers to previously described pitfalls and limitations, but it might raise a different level of concern. The use of chondrocytes genetically transfected to increase the expression of BMP-7 inoculated into a fibrin–collagen scaffold provided better histological results as compared with controls (rabbit model) [18].

TERM applications have not only been attempted in focal defects but also in global joint degeneration, i.e. arthritis. Joint replacement using biological tissue modified using TERM principles to mimic osteochondral tissue has been attempted [172]. In addition, the use of synthetic materials (e.g. ceramics) enhanced by MSCs aiming at future application in patients presently referred to fusion or total ankle arthroplasty has been evaluated [173].

**Figure 8.**
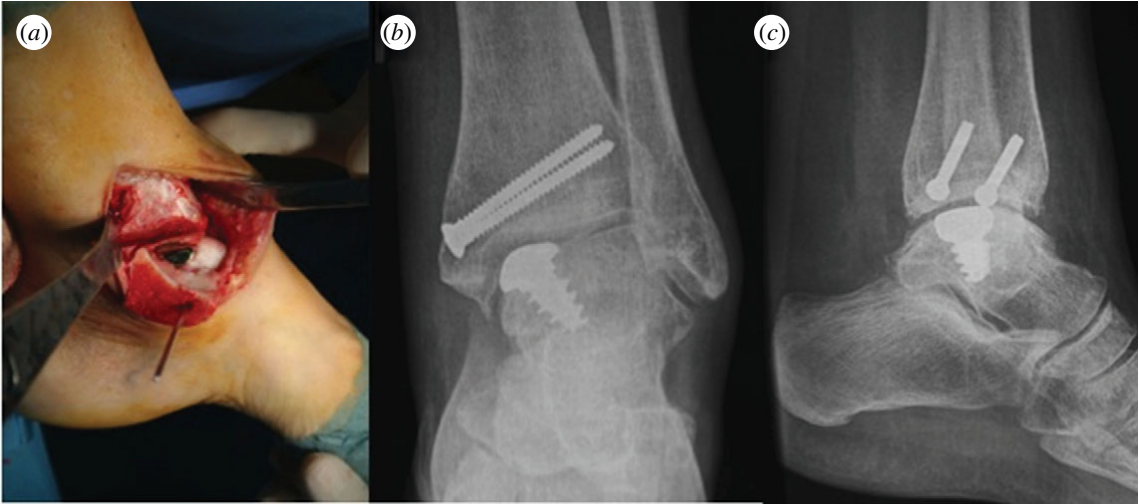
(*a*) Per-operative photograph of Hemicap ankle implant after tibial osteotomy and control X-ray in (*b*) frontal and (*c*) lateral views at 1 year follow-up.

Concerning focal defects, a non-biological solution developed by van Dijk's group [174] presented promising results by means of contoured focal metallic replacement (figure 8), despite the lack of mid- to long-term follow-up in larger series.

An important issue regarding the applications of biomaterials is the implant–tissue interface. Because of the geometric complexity of the ankle and the relative thickness of its cartilage, the use of focal resurfacing implants to treat talar OCDs, as well as biomaterials, presents challenges with regard to implant/biomaterial design, selection and surgical placement [175]. Considering the basic principles of TERM, besides biological conditions, ankle biomechanics must be taken into account [91] since it is a more congruent joint compared with the knee [176]. A congruent joint surface, for example the ankle, is usually covered with thinner hyaline cartilage compared with incongruent ones that possess thicker cartilage, for example in the knee. The diminishing of articular congruence produces higher contact pressure per joint area. Higher loss of congruence or malalignment will lead to growing contact pressure with all its implications [91,177,178]. Injured subchondral bone, as in OCDs, is less effective in supporting the overlying cartilage, and this might be one of the reasons explaining the greater difficulty for cartilage repair in these situations [179,180].

Becher *et al.* [181] measured contact stress redistribution in the human knee after implantation of a metallic resurfacing cap, and reported elevated contact stresses associated with device implants. Also, Custers *et al.* [182] stated that implants seem to cause considerable degeneration of the directly articulating cartilage in the knee. In the case of biomaterials, owing to their biocompatibility, integration into the surrounding cartilage is usually observed [183]. This way, the stress level changes on the joint are minor. However, the size and shape of the OCDs must be taken into account, to ensure that the biomaterial is as similar as possible, in order to completely fulfil the injured area.

## 3. Final considerations

The appropriate treatment for OCD repair is still controversial. The ideal technique would regenerate a tissue with biomechanical properties similar to normal hyaline articular cartilage, with reduced morbidity and costs. The excellent durability of results obtained by ACI or MACI over time is well established and contrasts sharply with the long-term results reported for bone-marrow-stimulating techniques (such as abrasion, drilling or microfractures).

A variety of biomaterials including polymers and ceramics have been proposed for regeneration of the cartilage of OCDs, and composite scaffolds (e.g. polymers combined with ceramics), especially if seeded with autologous cells and/or GFs, seem to improve biomechanical results.

Up to now only a few clinical trials on ankle healing have been described, whereas a scaffold approach to the treatment of knee chondral lesions has been largely used in clinical practice, with excellent or good clinical results largely documented in the literature. New approaches must be considered to talus osteocondral defects in order to improve restoration. Although there are particularities of such area, other biomaterials with significant results in knee OCDs may be applied to the ankle lesions.

TERM approaches are changing the paradigms of medicine and surgical practice. However, the success of these technologies at present and in future demands deep knowledge of native tissue biology and understanding of its repair mechanisms and response to injury, as well as the new biomaterials under consideration. Basic rules of biology and other ‘basic sciences’ (understanding basic only as fundamental, never as simple) must be well known by all surgeons since only in this way will they be able to understand, adapt and assist in the development of this knowledge to clinical practice.

TERM approaches have proven efficacy in clinical cases and problems which used selection criteria not previously solved by ‘conventional’ therapeutic repair and/or replacement options. However, undiscriminating use of any promising technique is one of the most effective ways to impair or even block its proper development.
